# *Ophiorrhizashiqianensis* (Rubiaceae), a new species from Guizhou, China

**DOI:** 10.3897/phytokeys.121.30570

**Published:** 2019-05-02

**Authors:** Lin-Dong Duan, Yun Lin, Zhen Lu

**Affiliations:** 1 Shaoyang University, Shaoyang 422004, Hunan, China Shaoyang University Shaoyang China; 2 Hunan Medication Vestibule School, Changsha 410208, Hunan, China Hunan Medication Vestibule School Changsha China

**Keywords:** Distyly, morphology, new taxon, taxonomy

## Abstract

A new species of the genus *Ophiorrhiza* L. (Rubiaceae), *Ophiorrhizashiqianensis* L.D.Duan & Yun Lin, is described, illustrated and photographed from Shiqian County, Guizhou Province, South-western China. This species was found growing at the side of streams in evergreen broad-leaved forests in mountains at elevations of 960–1100 m. The new species is morphologically similar to *Ophiorrhizahunanica* H.S.Lo, but differs from the latter by the glabrous, glabrescent or pilose stems, the 5–10 cm long subterranean stem internodes, the glabrous or pilose petioles, the 3–5 mm long stipules, the purple corolla lobes, the ca. 12 mm long style and included stigmas in long-styled flowers and the 3–4 × 8–10 mm, glabrous or glabrescent capsules.

## Introduction

The Rubiaceae genus *Ophiorrhiza* L. includes more than 300 species worldwide (WCSPF 2017) and is mainly distributed in tropical and subtropical Asia, Australia, New Guinea and the Pacific Islands (Darwin 1976, Chen and Taylor 2011). In China, *Ophiorrhiza* is represented by 70 species (Chen and Taylor 2011, Deng and Huang 2012, Wu et al. 2017a, b, Yang et al. 2018) and most of them are distributed in the region south of the Yangtze River, especially in the provinces of Yunnan and Guangxi (Lo 1999).

During three botanical explorations in Shiqian County, Guizhou Province, Southwest China, specimens of *Ophiorrhiza* were collected on the banks of streams in dense evergreen broad-leaved forests in valleys at elevations of 960–1100 m. After comparison of the newly collected specimens with material available in Chinese herbaria and careful consultation of literature, they were found to be most similar to *Ophiorrhizahunanica* H.S.Lo, but sufficiently different to qualify as a new species, hitherto not reported from any region in China. We therefore describe it as new, under the name *Ophiorrhizashiqianensis* L. D. Duan & Yun Lin.

## Materials and methods

Three field expeditions were carried out in Shiqian County, Guizhou Province, Southwest China in August 2011 and in March and May 2014 and a total of 30 mature individuals from the type locality (latitude 27°19'01.33"N, longitude 108°00'25.76"–108°00'35.76"E) were collected. All morphological measurements were performed on dried and fresh specimens. For the identification of specimens, relevant literature was used (Chen and Taylor 2011, Chen 2004, Deng and Huang 2012, Duan et al. 2014, Lin et al. 2017, Lo 1990, 1999, Wu et al. 2017a, b, Yang et al. 2018). The specimens were compared with herbarium material (about 5,000 specimens of the genus *Ophiorrhiza*) available at the herbaria CDBI, CSFI, GXMI, GZAC, GZTM, HGAS, HIB, HNNU, IBK, IBSC, IMC, KUN, LBG, NAS, PE, SZ and SYS (acronyms follow Fu 1993, Thiers continuously updated). The morphological characteristics of *Ophiorrhizashiqianensis* were determined using a stereo-trinocular microscope (Nikon SMZ1000) integrated camera system (Nikon DXM1200F). We used NTS-Elements D3.1 (Nikon Instruments Inc.) to make measurements.

## Taxonomy

### Ophiorrhiza shiqianensis

Taxon classificationPlantaeGentianalesRubiaceae

L.D.Duan & Yun Lin sp. nov.

urn:lsid:ipni.org:names:77196565-1

Figs 1 , 2 , 4A–E

#### Diagnosis.

Similar to *Ophiorrhizahunanica* H. S. Lo based on stems, leaves and capsules; differing from it by the stems which are glabrous, glabrescent or pilose, the 5–10 cm long subterranean stem internodes, the glabrous or pilose petioles, the 3–5 mm long stipules, the purple corolla tube and lobes, the ca. 12 mm long styles and the included stigmas in long-styled flowers, the 3–4 mm × 8–10 mm, glabrous or glabrescent capsules [vs. stems densely villose, subterranean stem internodes 1–2 cm long, petioles villose, stipules 5–15 mm long, corolla tube purple and corolla lobes white, style 15–17 mm long and stigmas exserted in long-styled flowers, capsules 5–6 mm × 10–12 mm, densely villose in *Ophiorrhizahunanica* (Figs 3, 4F)].

**Figure 1.**
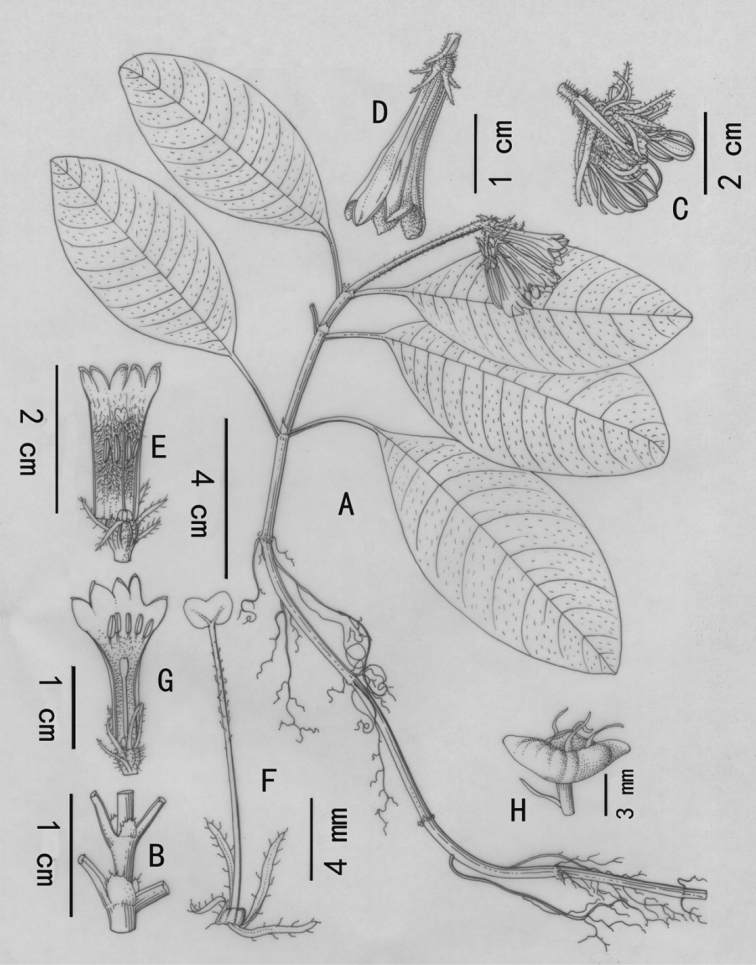
*Ophiorrhizashiqianensis*. **A** habit of flowering plant **B** section of stem, showing stipules **C** inflorescence **D** flower **E** long-styled flower **F** pistil in a long-styled flower **G** short-styled flower **H** capsule **A–F** from *L.D. Duan, Z. Lu & Q. Lin 5805***G** from *L.D. Duan, Z. Lu & Q. Lin 5808***H** from *L.D. Duan, Z. Lu & Q. Lin 5809*.

**Figure 2.**
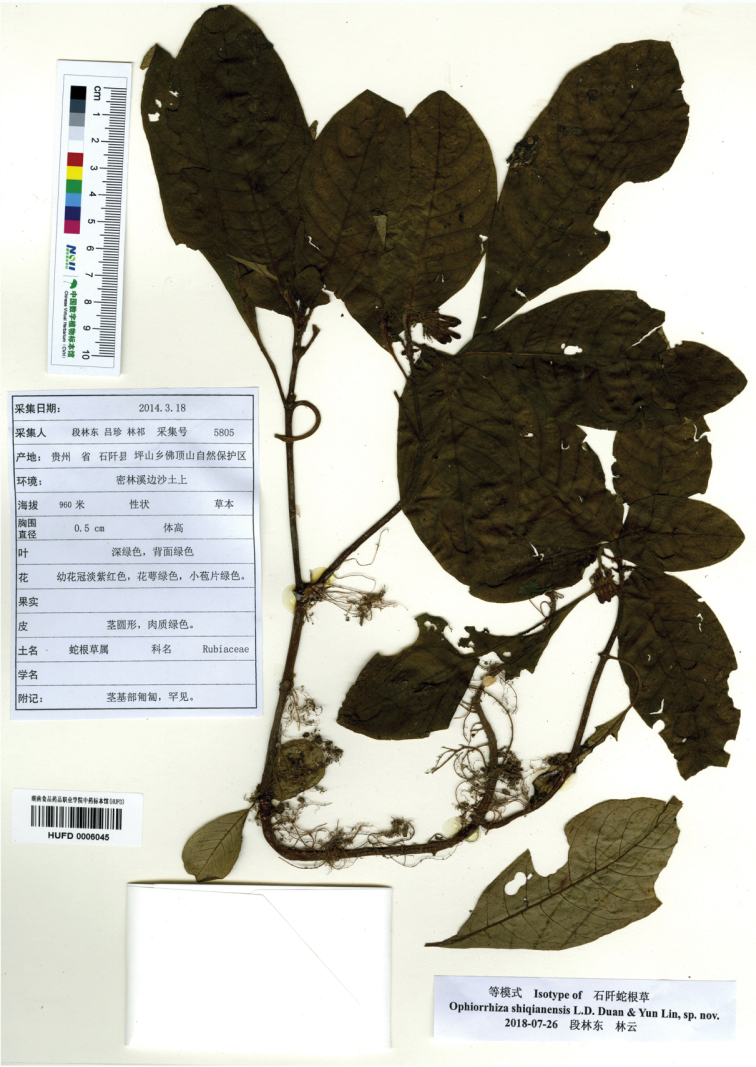
*Ophiorrhizashiqianensis.* Isotype, showing subterranean stem internodes 5–10 cm long and inflorescences 2- to 10-flowered.

**Figure 3.**
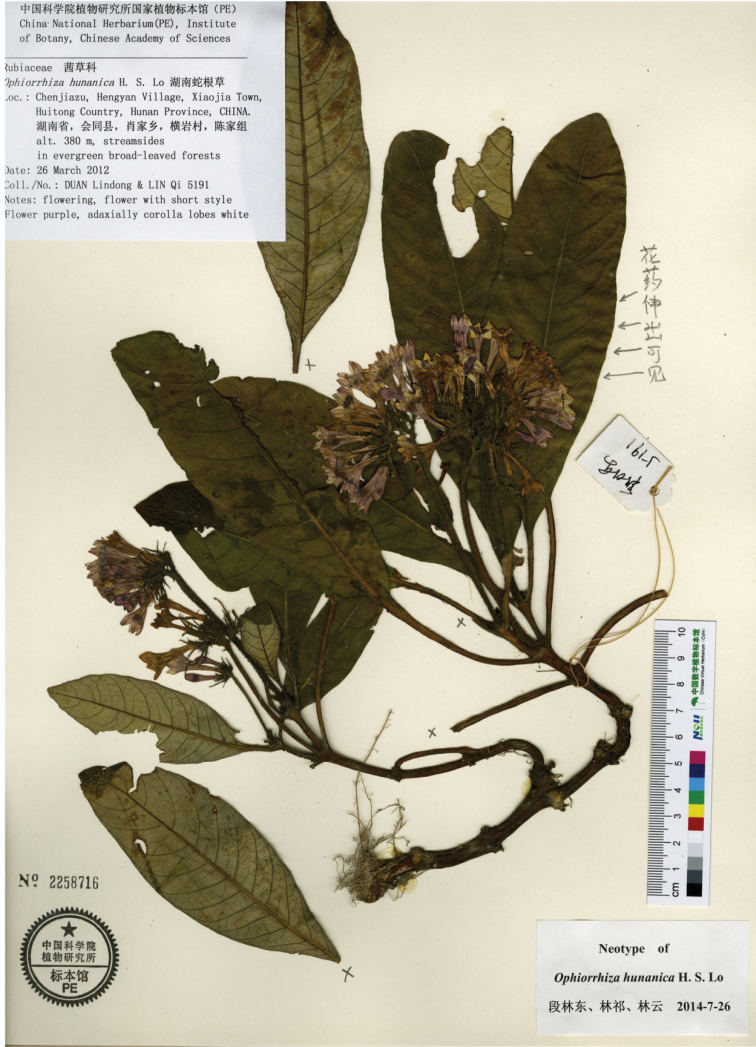
*Ophiorrhizahunanica*. Neotype (designated by Duan et al. 2014), showing subterranean stem internodes 1–2 cm long and inflorescences 5- to many-flowered.

**Figure 4.**
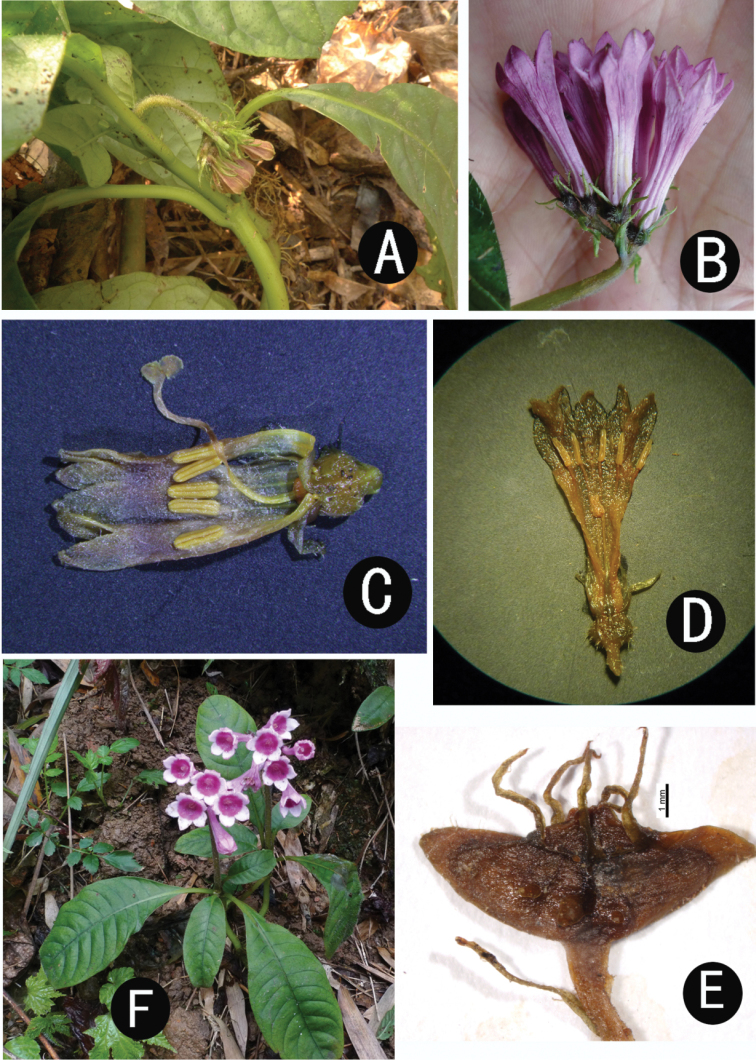
*Ophiorrhizashiqianensis* (**A–E**) and *O.hunanica* (**F**). **A** Habit of flowering plant **B** Inflorescence **C** long-styled flower **D** short-styled flower **E** capsule **F** habit of flowering plant, showing white corolla lobes.

#### Type.

CHINA. Guizhou: Shiqian County, Pingshan Town, Fudingshan Nature Reserve, at sides of streams in dense evergreen broad-leaved forest in valley at 960 m elevation, 18 March 2014 (fl), *L.D. Duan, Z. Lu & Q. Lin 5805* (holotype: PE!, Herb. Bar. Code No. 02232812; isotypes HUFD! (=Herbarium, Hunan Food and Drug Vocational College, Hunan, China), HUSY! (=Herbarium, Shaoyang University, Hunan, China) IBSC!, K!, KUN!, PE!).

#### Description.

Perennial herbs, 10–30 cm tall, repent at base. Stems erect, fleshy, green, brown to black after drying, glabrous, glabrescent or pilose; subterranean stems glabrous with 5–10 cm long internodes. Leaves: petioles 1.5–4.0 cm long, glabrous or pilose; blades papery after drying, elliptic or obovate-elliptic, 7–17 cm × 3–7.5 cm, adaxially pilose, abaxially pilose on veins, base cuneate, apex obtuse to subacute; secondary veins 9–11 pairs; stipules often persistent, ovate to ovate-lanceolate, 3–5 mm long, ciliate. Inflorescence cymose, terminal, 2- to 10-flowered, densely villose, pendulous; peduncle 2.5–4 cm long when flowering, 8–10 cm long when fruiting, arching, densely villose. Bracts linear-lanceolate, 9–10 mm long, ciliate; bracteoles linear, 4–5 mm long, ciliate. Flowers distylous, pedicels 1–2 mm long, densely covered with hairs. Calyx with hypanthium compressed-turbinate, 2.5–3 mm long, 5-ribbed, densely covered with hairs; lobes 5, lanceolate-linear, 5–6 mm long, ciliate. Corolla tube and lobes purple, funnel-form, outside glabrous, inside pubescent; tube 1.7–1.8 cm long; lobes 5, ovate-triangular, 4–6 mm long, apex rostrate. Stamens 5, inserted near throat and exserted at anthesis in short-styled flowers or inserted below middle of corolla tube and included at anthesis in long-styled flowers; filaments 1.5–2 mm long in short-styled or ca. 1 mm long in long-styled flowers; anthers ca. 2.5 mm long, dorsifixed. Ovary 2-celled, ovules numerous in each cell; style ca. 12 mm long in long-styled or 7–7.5 mm long in short-styled flowers; stigmas 2, linear in short-styled or subcapitate in long-styled flowers, included in both morphs. Capsules purple, mitriform, strongly laterally compressed, 3–4 mm × 8–10 mm, pilose or glabrescent. Seeds numerous.

#### Phenology.

Plants were observed in full bloom on 18 March 2014 and with ripe fruits on 12 May 2014 and neither flowers nor fruits were seen on 12 August 2011. It can be expected that the flowering time of the new species is from March to April and that fruiting time is from April to June.

#### Habitat.

The species grows on the banks of streams in dense broad-leaved forest in valleys at elevations of 960–1100 m.

#### Distribution.

*Ophiorrhizashiqianensis* is only known from two localities in Shiqian County, northeast Guizhou Province, southwest China, notably: Fudingshan Nature Reserve, Pingshan Town and Nishan Village, Ganxi Town.

#### Etymology.

*Ophiorrhizashiqianensis* is named after its type locality, Shiqian County, northeast Guizhou Province, southwest China.

#### Vernacular name.

Shi qian she gen cao in Chinese Pinyin.

#### Preliminary conservation status.

*Ophiorrhizashiqianensis* is only known from four collections in two locations, Fudingshan Nature Reserve (well protected, 152 km^2^) and Nishan Village (ca. 50 km^2^). It comprises about 200 individuals (criteria D1 ≤ 250) growing in ten populations. This new species can be assessed as Endangered (EN) according to the IUCN Red List Categories and Criteria (IUCN 2001, 2012). However, it is possible that more populations could be found in similar habitats of mountain areas of Zhenyuan, Yuqing and Shibing Counties in northeast Guizhou. With limited fieldwork at present, we would temporarily consider this new species to be **Vulnerable (VU) based on criteria D1 and D2.**

#### Additional specimens of *Ophiorrhizashiqianensis* (paratypes).

CHINA. Guizhou: Shiqian County, Ganxi Town, Nishan Village, Niujinshan, stream-sides in dense evergreen broad-leaved forest in valley at 1100 m elevation, 12 August 2011 (sterile), *L. D. Duan, Z. Lu & Q. Lin 5356* (HUFD!, HUSY!, PE!); Pingshan Town, Fudingshan Nature Reserve, stream-sides in dense evergreen broad-leaved forest in valley at 960 m elevation, 18 March 2014 (fl), *L. D. Duan, Z. Lu & Q. Lin 5808* (HUFD!, HUSY!, PE!); same locality, 12 May 2014 (fr), *L. D. Duan, Z. Lu & Q. Lin 5809* (HUFD!, HUSY!, IBSC!, K!, KUN!, PE!).

#### Critical note.

The new species most resembles *Ophiorrhizahunanica*. Detailed morphological differences between the two species are given in Table 1.

**Table 1.** Comparison of morphological characteristics between *Ophiorrhizashiqianensis* and *O.hunanica*.

Characters	* O. shiqianensis *	* O. hunanica *
Plant	repent at base	procumbent or repent at base
Stem	glabrous, glabrescent or pilose; subterranean stem internodes 5–10 cm long	villose; subterranean stem internodes 1–2 cm long
Leaf	petiole 1.5–4.0 cm long, glabrous or pilose; blade 7–17 cm long; stipules 3–5 mm long	petiole 1–6 cm long, villose; blade 7–23 cm long; stipules 5–15 mm long
Flower	2- to 10-flowered; peduncle 2.5–4 cm long when flowering, 8–10 cm long when fruiting; corolla tube and lobes purple; style c. 12 mm long and stigmas included in long-styled flowers	5- to many-flowered; peduncle 3–8 cm long when flowering, 12–15 cm long when fruiting; corolla tube purple and lobes white; style 15–17 mm long and stigmas exserted in long-styled flowers
Capsule	3–4 mm × 8–10 mm, glabrous or glabrescent	5–6 mm × 10–12 mm, densely villose

## Supplementary Material

XML Treatment for Ophiorrhiza shiqianensis
